# A Rapid and Sensitive *Salmonella* Biosensor Based on Viscoelastic Inertial Microfluidics

**DOI:** 10.3390/s20092738

**Published:** 2020-05-11

**Authors:** Lan Yao, Lingyan Zheng, Gaozhe Cai, Siyuan Wang, Lei Wang, Jianhan Lin

**Affiliations:** Key Laboratory of Agricultural Information Acquisition Technology, Ministry of Agriculture and Rural Affairs, China Agricultural University, Beijing 100083, China; ylan2014@cau.edu.cn (L.Y.); Lingyanzheng@cau.edu.cn (L.Z.); gaozhe@cau.edu.cn (G.C.); wangsiyuan@cau.edu.cn (S.W.); wanglei123@cau.edu.cn (L.W.)

**Keywords:** microfluidic biosensor, viscoelastic inertial microfluidics, particle separation, enzyme catalytic colorimetry, bacteria detection

## Abstract

*Salmonella* is a main cause of foodborne illnesses and rapid screening of *Salmonella* is the key to prevent *Salmonella* outbreaks, however available detection methods either require a long time, or need complex pretreatment, or have low sensitivity. In this study, a microfluidic biosensor was developed for *Salmonella* detection using viscoelastic inertial microfluidics for separating magnetic bacteria from unbound magnetic nanoparticles (MNPs) and enzyme catalytic colorimetry for amplifying biological signals. The polyclonal antibodies and horseradish peroxidase (HRP) modified MNPs were first used to specifically capture *Salmonella* to form magnetic HRP-bacteria. Both magnetic HRP-bacteria and unbound MNPs were magnetically separated from background and resuspended in viscoelastic polyvinylpyrrolidone solution as sample flow. When sample flow was injected with polyvinylpyrrolidone sheath flow into a T-shaped microchannel, larger-sized magnetic HRP-bacteria could penetrate the sample flow, however smaller-sized MNPs remained in the sample flow due to weaker inertial lift force and elastic lift force, resulting in continuous-flow separation of magnetic HRP-bacteria. Finally, magnetic HRP-bacteria were collected and concentrated to catalyze tetramethyl benzidine, and absorbance was measured to determine the bacteria. This biosensor was able to detect *Salmonella* as low as 30 CFU/mL in 1 h and featured the advantages of shorter time due to a one-step immunoreaction, easier extension due to only one antibody and one label, and lower cost due to less expensive materials.

## 1. Introduction

Food safety has attracted more and more concerns globally, and foodborne illnesses are mainly caused by foodborne pathogenic bacteria. A key to prevent the outbreaks of foodborne illnesses is early screening of bacteria contaminated foods. Currently available methods, such as polymerase chain reaction [1,2,3], enzyme-linked immunosorbent assay [4,5,6] and culture plating, have been applied for bacteria detection, however they have limitations of either complex DNA extraction, or relatively low sensitivity, or long detection time. Therefore, simple, rapid and sensitive methods for bacteria detection are urgently needed to ensure food safety.

Due to the complexity of the food matrix, target bacteria often need to be separated from the complex background to minimize non-specific reaction and improve sensitivity [7]. Immunomagnetic separation has been widely used for specific separation of various biological targets from different samples in the past decades [8,9,10], since it can not only purify the targets to reduce the background noises, but also concentrate the targets in a small volume of buffer solution to improve the detection sensitivity. Magnetic nanoparticles (MNPs) modified by biological recognition components, such as antibodies and aptamers, are first used to specifically identify and capture target bacteria in food samples, and the target bacteria conjugated with the MNPs (magnetic bacteria) are then oriented at the presence of an external magnetic field, leading to specific separation of target bacteria from sample background [11,12]. Although this conventional magnetic separation has shown its great merits of good specificity and high separation efficiency, there still is a big challenge to directly use the MNPs as detection signal, which could obviously reduce the detection cost, save the detection time and simplify the detection procedure, since the magnetic bacteria are co-existing with the unbound MNPs. 

In recent years, several studies have been attempted to separate the magnetic bacteria from the unbound MNPs. Some of them were based on the size difference. At present, most commercially available MNPs usually have the diameter of 20–200 nm, while foodborne pathogenic bacteria are basically 1–3 μm, which are much larger than the MNPs. Shim [13] et al. developed a typical filtration method combined with immunomagnetic separation to detect *Salmonella* in vegetables. After the target bacteria were separated using magnetic separation, the magnetic bacteria were further separated from unbound MNPs using filtration membrane, resulting in the trap of magnetic bacteria on the membrane, whose color was measured to determine the target bacteria. This method was able to detect *Salmonella* as low as 100 cells/g in 45 min, however it might suffer from clogging and inaccurate separation. Another interesting study was proposed based on size-based separation by Lee et al. [14] using a 3D-printed microfluidic device to separate the magnetic bacteria from the unbound MNPs in a helical channel with trapezoid cross-section. The other studies were based on the magnetic response difference. One target bacterium is often conjugated with multiple MNPs [15], making the magnetic bacterium have a stronger magnetic response in the magnetic field than one unbound MNP. A couple of interesting studies on magnetophoretic chromatography with immunomagnetic separation were reported by Kwon et al. [16,17] to detect pathogenic bacteria. After the immune magnetic nanoclusters (MNCs) were used to magnetically separate the target bacteria in milk, the viscous polyethylene glycol (PEG) solution and the mixture of the magnetic bacteria and the unbound MNCs were successively sucked into the pipette tip and two layers were formed. After the strong magnet was placed under the tip, the magnetic bacteria moved from the buffer to PEG solution because the downward magnetic force was larger than the upward buoyancy, however the unbound MNCs remained in the PEG solution due to the smaller magnetic force. Finally, the bacteria were determined by measuring the color change of the interface of the solutions. This method was able to detect *Salmonella* as low as 100 CFU/mL, however it required very precise operation. 

In the past decades, particle separation technologies based on inertial microfluidics have received an increasing attention due to their smaller sample consumption, shorter separation time, and easier integration [18,19,20,21,22]. The particles with different sizes are often separated in the microfluidic chips based on the difference of inertial force on the particles, which is proportional to the mass (size) of the particles [23]. Since only the particles with larger sizes can experience enough force to change their moving trajectory, inertial force is usually used to separate the particles with the size of >3 μm [24], however both the bacteria and the MNPs are too small and not susceptible to inertial force. Recently, coupling enhancement of viscoelastic force and inertial force was demonstrated to successfully separate the particles with submicron size from viscoelastic fluid [25,26]. Liu et al. [27] used polyethylene oxide (PEO) as viscoelastic fluid to separate the particles with different sizes in a T-shaped straight channel, and verified that the particles with the size of 100 nm could be successfully separated from those with the size of 500 nm. 

**Figure 1:**
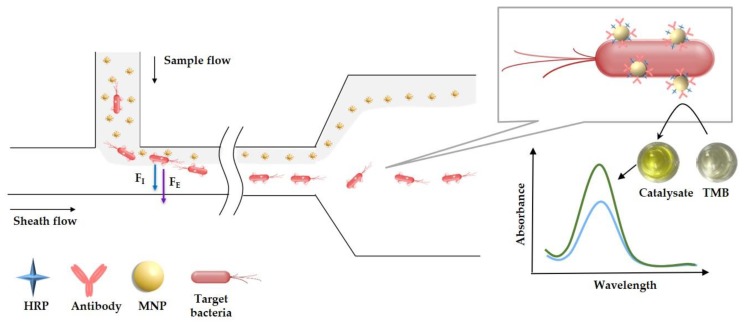
Schematic of the microfluidic biosensor based on viscoelastic particle separation and enzyme catalytic colorimetry.

In this study, we developed a novel microfluidic biosensor for detection of *Salmonella* typhimurium based on viscoelastic particle separation for isolating the magnetic bacteria from the unbound MNPs and enzyme catalytic colorimetry for amplifying and measuring the biological signals. As shown in Figure 1, the MNPs modified with anti-*Salmonella* polyclonal antibodies (PAbs) and horseradish peroxidase (HRP) were first conjugated with *Salmonella* typhimurium to form the bacteria-MNP-HRP complexes (magnetic HRP-bacteria). After magnetic separation, both the magnetic HRP-bacteria and the unbound MNPs were concentrated in the viscoelastic polyvinyl pyrrolidone (PVP) solution as sample flow. Then, the sample flow and the sheath flow (the same PVP solution) were simultaneously injected into the T-shaped separation microchannel, and combined force of elastic lift force (***F_E_***) and inertial lift force (***F_I_***) would intercept the smaller-sized MNPs and allow the penetration of the larger-sized magnetic HRP-bacteria. After the magnetic HRP-bacteria were collected at the central outlet, they were used to catalyze tetramethyl benzidine (TMB) and the color development was terminated with sulfuric acid, followed by measuring the absorbance to determine the concentration of the bacteria. 

## 2. Materials and Methods

### 2.1. Preparation of Bacteria

*Salmonella* typhimurium (ATCC14028) was used as target bacteria while *Listeria monocytogenes* (ATCC 13932), *Escherichia coli O157:H7* (ATCC 43888) *Staphylococcus aureus* (CICC10001), *Salmonella* derby, *Salmonella* enteritidis, *Salmonella* mbandaka, and *Salmonella* meleagridis were used as non-target bacteria. The bacteria were first cultured in the LB medium (Aoboxing Biotech, Beijing, China) at 37 °C for 12–16 h with shaking at 180 rpm, and then were serially diluted by phosphate buffer saline (PBS, 10 mM, pH 7.4, Sigma Aldrich) to obtain the bacteria at the concentrations from 10^1^ to 10^6^ CFU/mL.

### 2.2. Fabrication of the Microfluidic Chip

The microfluidic chip plays the most important role in separation of the magnetic HRP-bacteria from the unbound MNPs. It mainly included an asymmetric T-shaped separation microchannel with the length of 31 mm, the width of 50 μm and the depth of 25 μm, which was fabricated using soft lithography. More details can be found in Figure S1 in the Supplementary Materials. The microchannel was connected with two 4 mm long side-branch channels for injecting the sample flow and the sheath flow, respectively. The end of each side-branch channel is a 600 μm wide expansion region with 5 rows of small rods (diameter: 50 μm) for avoiding the blocking of the microchannel by the large particles in food samples. The end of the main-branch channel is an 800 μm wide expansion region for increasing the separation distance and observing the trajectories of the particles. The particle motions at the expansion region and other regions of the microchannel were visualized and recorded using an inverted fluorescent microscope (Eclipse Ti, Nikon, Kyoto, Japan) with a CCD camera at the rate of around 15 frames per second. Fluorescent and bright-field lights were simultaneously used to visualize both the fluorescent particles (appearing in green) and the channel edges (appearing in black). 

### 2.3. Modification of the MNPs

The MNPs modified with the polyclonal antibodies and horseradish peroxidase (Solarbio, Beijing, China) were used to capture the target bacteria and amplify the biological signals. HRP was first modified with biotin using the biotinylation kit (Elabscience, Wuhan, China) according to the manufacturer’s protocol. The biotinylated HRP with the concentration of 1 mg/mL was stored at −20 °C with glycerol. Prior to test, 10 µL of the streptavidin conjugated MNPs (Fe concentration: 1 mg/mL, Diameter: 100 nm, MHS-150-10, Ocean Nanotech, Dunedin, FL, USA) were incubated with 1 µL of the biotinylated PAbs against *Salmonella* typhimurium (Concentration: 2 mg/mL, Abcam, Cambridge, MA, USA) and 0.5 µL of HRP at 15 rpm for 45 min and magnetically separated using a magnetic separator (MS0406, Aibit Biotech, Wuxi, China) for 2 min to remove the excessive PAbs and HRP. Finally, the MNPs were resuspended with 1 mL of PBS containing 1% BSA to obtain the PAb-MNP-HRP conjugates (immune HRP-MNPs), which were stored at 4 °C for further use.

### 2.4. Separation of the Magnetic HRP-Bacteria

For separation of the magnetic HRP-bacteria, both polyvinyl pyrrolidone and polyethylene oxide were purchased from Sigma Aldrich (St. Louis, MO, USA) and prepared in the deionized water (1%, w/v, 18.2 MΩ·cm, Advantage 10, Millipore, Billerica, MA, USA) as the viscoelastic solution. For parameter optimization, the fluorescent polystyrene (PS) microspheres with the sizes of both 2.2 µm and 100 nm purchased from VDO Biotech (Suzhou, China) were prepared in the PVP solution with the concentration of 0.05% (w/v) for simulating the magnetic HRP-bacteria and the unbound MNPs. After the magnetic HRP-bacteria were formed, they were resuspended in 50 µL of the PVP solution. To prevent the particles from aggregating during the separation, Tween-20 (0.5%, v/v, Amresco, Solon, OH, USA) was added into both the sample flow and the sheath flow. 

The sample flow and the sheath flow were injected into the microchannel using two precision syringe pumps (11Elite, Harvard Apparatus, FL, USA) with different flow rates. The separation of the fluorescent PS microspheres was observed using the fluorescent inverted microscope. The fluorescent trajectories were recorded using long exposure time (up to 600 ms). The experimental results were analyzed using the microscope’s software NIS-Elements AR 2.30.

### 2.5. Detection of the Target Bacteria

The target bacteria were first specifically separated from the background using immunomagnetic separation (45 min), and the magnetic HRP-bacteria were then separated from the unbound MNPs using viscoelastic particle separation (10 min) and finally detected using enzymatic catalysis colorimetry (5 min). First, 10 µg of the immune HRP-MNPs were incubated with 500 µL of the sample containing different concentrations (10^1^–10^6^ CFU/mL) of *Salmonella* typhimurium at 15 rpm for 45 min to form the magnetic HRP-bacteria. After that, the mixture of the magnetic HRP-bacteria and the unbound MNPs was resuspended in the viscoelastic solution with 0.5% Tween-20. Then, the magnetic HRP-bacteria were separated from the unbound MNPs using the microfluidic chip. After magnetic separation to remove the viscoelastic solution, the magnetic HRP-bacteria were resuspended in 100 µL of PBS and 50 µL of the suspension was pipetted into the microplate. 100 µL of tetramethyl benzidine from Solarbio was added into the microplate and incubated for 5 min, followed by adding 100 µL of 1 M H_2_SO_4_ to terminate the catalytic reaction. Finally, the catalysate was measured using Infinite M200 PRO (Tecan, Männedorf, Switzerland) and the absorbance at the characteristic wavelength of 450 nm was used to determine the concentration of the bacteria.

## 3. Results and Discussion

### 3.1. Mechanism of Viscoelastic Particle Separation

The mechanism of the particle separation in the viscoelastic solution was illustrated in Figure 1. The mixture of different sizes (~2 µm and ~100 nm) of particles was first squeezed by the sheath flow into a thin layer near the top wall of the microchannel at the T-junction. The inherent elastic lift force (***F_E_***) and inertial lift force (***F_I_***) were induced in the viscoelastic fluid to push the particles away from the sidewall in the elongated rectangular main branch. $F_{E}$ can be expressed as:

(1) $$F_{E}\sim r_{p}^{3}\nabla N_{1}\sim r_{p}^{3}W_{i}{\dot{\gamma }}^{2}$$

where, $r_{p}$ is the particle’s radius, $N_{1}$ is the first normal stress difference, *Wi* is the Weissenberg number, and γ is the average shear rate. It increases with $W_{i}$ and can push the particles to move toward the regions with lower shear rate, i.e., the centerline in the rectangular microchannel. For the particles near the sidewall, $F_{I}$ can be expressed as:

(2) $$F_{I}\sim \rho V_{m}^{2}r_{p}^{6}/w^{4}$$

where, *ρ* is the fluid density, $V_{m}$ is the maximum fluid velocity, and *w* is the width of the microchannel. It increases with $V_{m}$ and can push the particles to move away from the sidewall. In this study, both $F_{E}$ and $F_{I}$ work together to move the particles toward the channel’s center. 

### 3.2. Selection of the Viscoelastic Fluid

**Figure 2:**
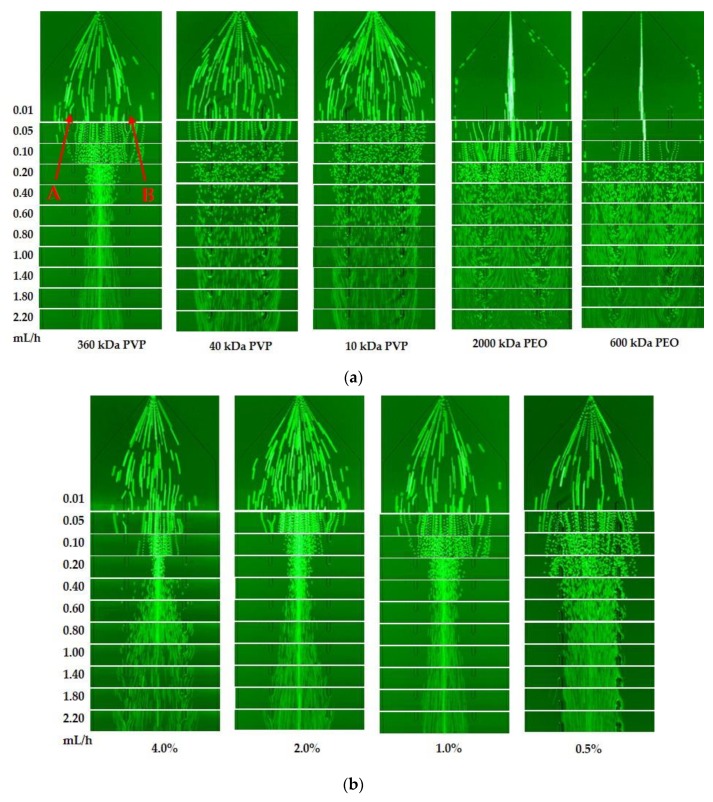
(**a**) Images at the expansion of the main-branch for the aggregation of the particles with the diameter of 2.2 µm in 600 kDa polyethylene oxide (PEO), 2000 kDa PEO, 10 kDa polyvinyl pyrrolidone (PVP), 40 kDa PVP and 360 kDa PVP at the velocity range from 0.01 mL/h to 2.20 mL/h; (**b**) images at the expansion of the main-branch for the aggregation of the particles with the diameter of 2.2 µm in different concentrations of 360 kDa PVP (0.5%, 1.0%, 2.0% and 4.0%) at the velocity range from 0.01 mL/h to 0.80 mL/h.

The viscoelastic fluid is the key to separation of the magnetic HRP-bacteria from the unbound MNPs. Therefore, two reported viscoelastic fluids (PEO and PVP) with different molecular weights and concentrations were compared to select a better viscoelastic fluid. The fluorescent PS microspheres with the diameter of 2.2 µm were used to mimic the magnetic HRP-bacteria and dissolved in 600 kDa PEO (1%, w/v), 2000 kDa PEO (1%, w/v), 10 kDa PVP (1%, w/v), 40 kDa PVP (1%, w/v) and 360 kDa PVP (1%, w/v), respectively, followed by separation at the velocity from 0.01 mL/h to 2.20 mL/h. As shown in Figure 2a, the PS microspheres were focused in the centerline (between the point A and B) in both PEO fluids only at a low velocity (<0.05 mL/h), however some microspheres were found at both sidewalls of the microchannel, indicating that PEO was not the ideal viscoelastic fluid. Besides, the microspheres were not focused in both 40 kDa PVP and 10 kDa PVP, but focused well in 360 kDa PVP. Thus, the optimal viscoelastic fluid of 360 kDa PVP was used in this study. 

The velocity is another key to separation of the magnetic HRP-bacteria. Thus, different velocities was applied for separation of the PS microspheres in 360 kDa PVP. As shown in Figure 2a, when the velocity increased from 0.01 to 0.1 mL/h, the microspheres were gradually aggregated into the middle channel and no microspheres were found near the sidewalls due to the pushing of the elastic and inertial lift forces from the sidewalls towards the center. When the velocity increased from 0.2 to 0.8 mL/h, the microspheres became more and more focused in the center of the microchannel because the increasing inertial lift force pushed the microspheres harder towards the center. However, when the velocity kept increasing to 2.20 mL/h, the microspheres started to disperse probably because the drag force on the microspheres in the longitudinal direction increased with the velocity and became dominant compared to the elastic and inertial forces in the lateral direction. Thus, the velocity from 0.2 to 1.8 mL/h was suitable to focus the microspheres in 360 kDa PVP.

To further investigate the impact of the concentration of the viscoelastic fluid on the separation of the particles, different concentrations (0.5%, 1%, 2% and 4%, w/v) of 360 kDa PVP were used to separate the PS microspheres. As shown in Figure 2b, the microspheres were well focused at the concentrations of 1% and 2% with the velocity ranging from 0.2 mL/h to 0.8 mL/h, and at the concentration of 4.0% with the velocity of 0.2 mL/h, while they were not focused at the concentration of 0.5%. To trade off the separation speed and effect, the optimal concentration of 1% for 360 kDa PVP was used in this study. 

### 3.3. Optimization of the Velocity of Sample Flow and Sheath Flow

**Figure 3:**
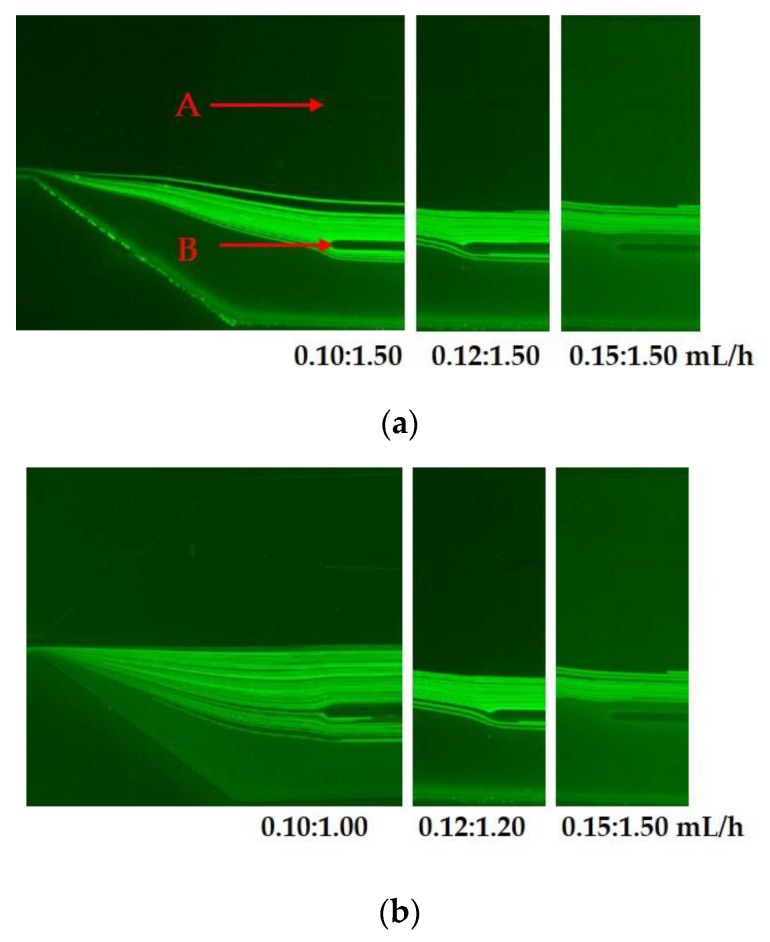
(**a**) Images at the expansion of the main-branch for the separation of the 2.2 µm particles (in the middle of the microchannel) and the 100 nm particles (near the bottom sidewall of the microchannel) in 360 kDa PVP at different velocity ratios (α = 15, 12.5 and 10); (**b**) images at the expansion of the main-branch for the separation of the 2.2 µm particles (in the middle of the microchannel) and the 100 nm particles (near the bottom sidewall of the microchannel) in 360 kDa PVP at different sheath flow rates (1, 1.2 and 1.5 mL/h) and the same velocity ratio of 10.

The velocities of the sample flow and the sheath flow play an important role in the separation of the magnetic HRP-bacteria. The fluorescent PS microparticles with the diameters of 2.2 µm and 100 nm were used to mimic the magnetic HRP-bacteria and the unbound MNPs, respectively. Different velocity ratios of the sample flow to the sheath flow (α = sheath flow/sample flow: α = 1.50/0.10 mL/h = 15, α = 1.50/0.12 mL/h = 12.5, and α = 1.50/0.15 mL/h = 10) were compared to separate the microspheres. As shown in Figure 3a, part of the 2.2 µm microspheres were not focused in the central outlet (between point A and B) when the velocities of the sample flow were 0.10 and 0.12 mL/h, and all of them were focused in the central outlet when the velocity was 0.15 mL/h. For the 100 nm microspheres, they were mostly focused in the bottom side of the microchannel. Thus, the optimal velocity ratio α = 10 of sheath flow to sample flow was used in this study. 

Furthermore, different flow rates with the same velocity ratio of 10 (sheath flow/sample flow = 1/0.1, 1.2/0.12 and 1.5/0.15 mL/h) were compared to separate the microspheres with the diameter of 2.2 µm and 100 nm. As shown Figure 3b, the microspheres were focused in the central outlet only when the velocity of the sample flow was 0.15 mL/h. The 100 nm microspheres were almost compressed on the bottom of the microchannel. Thus, the optimal velocities of the sheath flow and the sample flow were 1.5 and 0.15 mL/h, respectively.

### 3.4. Optimization of the PAb-to-HRP Ratio

**Figure 4:**
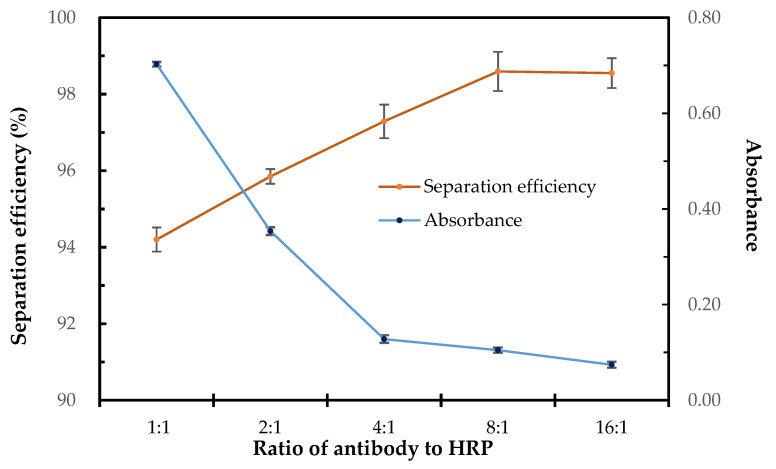
The separation efficiency and absorbance for the MNPs with different ratios of antibody to horseradish peroxidase (HRP) (N = 3).

The antibody and HRP modified on the MNPs have great impact on the sensitivity of this biosensor, since more antibodies modified on the MNPs might lead to higher capture efficiency and more HRP modified on the MNPs might lead to higher detection signal, however the total binding sites for immobilization of PAbs and HRP were fixed. Thus, it is crucial to optimize the ratio of PAbs to HRP. Different ratios of PAbs to HRP from 1:1 to 16:1 were applied to modify the MNPs. The modified MNPs were used to separate the target bacteria and the separation efficiency of the bacteria was determined using culture plating to evaluate the PAbs performance of the MNPs. Besides, the modified MNPs were also used to detect negative samples and the concentration of the catalysate was determined measuring the absorbance to evaluate the HRP performance of the MNPs. As shown in Figure 4, the separation efficiency of the target bacteria increased slightly from 94% to 98% when the ratio of PAbs to HRP changed from 1:1 to 8:1, indicating that the antibodies on the MNPs were sufficient for each ratio to separate the target bacteria. However, the absorbance of the catalysate decreased dramatically from 0.70 to 0.13 when the ratio of PAbs to HRP changed from 1:1 to 4:1 and kept decreasing to 0.07 when the ratio changed from 4:1 to 16:1. It indicated that the biotinylated HRP had strong binding ability to the streptavidin coated MNPs, and the amount of HRP modified on the MNPs was too much when the ratios of PAbs to HRP were 1:1 and 2:1,resulting in strong background signals. Thus, the optimal PAbs-to-HRP ratio of 4:1 was used in this study.

### 3.5. Detection of Salmonella Typhimurium in Pure Sample

**Figure 5:**
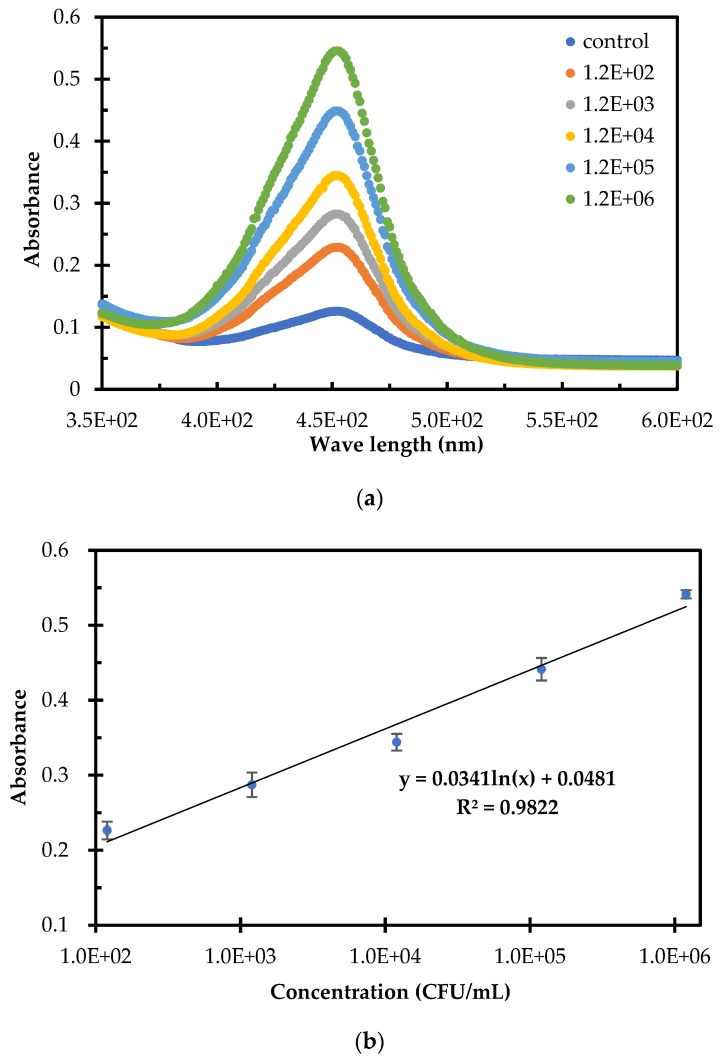

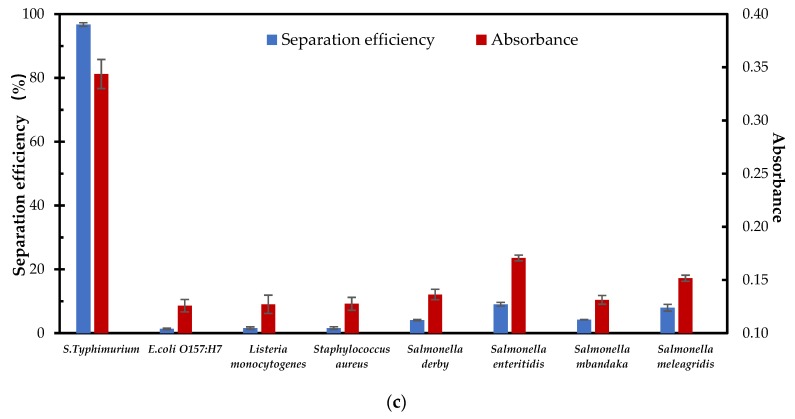
Detection of Salmonella Typhimurium in Pure Sample. (**a**) The absorbance spectra for different concentrations of *Salmonella* typhimurium in pure samples; (**b**) the linear relationship between the absorbance of catalysate and the concentration of *Salmonella* typhimurium in pure samples (N = 3); (**c**) Comparison of separation efficiency and absorbance of target bacteria and non-target bacteria (N = 3).

To evaluate this biosensor for detection of foodborne bacteria, three parallel tests on *Salmonella* typhimurium at different concentrations of 1.2 × 10^2^ to 1.2 × 10^6^ CFU/mL in the pure cultures and negative samples were conducted. The absorbance spectra for different concentrations of *Salmonella* typhimurium were shown in Figure 5a. When the concentration of the target bacteria changed from 1.2 × 10^2^ CFU/mL to 1.2 × 10^6^ CFU/mL, the absorbance of the catalysate increased obviously, indicating that more magnetic HRP-bacteria were formed and collected with more HRP to catalyze TMB. To further build up the calibration curve of this biosensor, the absorbance at the characteristic wavelength of 450 nm were plotted with the concentration of *Salmonella* typhimurium. As shown in Figure 5b, a good linear relationship between the absorbance (A) of the catalysate and the concentration (C) of *Salmonella* typhimurium was found and could be expressed as:

(3) A=0.0341×ln(C)+0.0481

Based on three times of signal-to-noise ratio, the detection limit of this biosensor was calculated as 30 CFU/mL. The high sensitivity of this biosensor might be due to the following aspects: (1) the efficient separation of the magnetic HRP-bacteria from the unbound MNPs to greatly reduce the background noises; (2) the effective amplification of the biological signals using the HRP; (3) the enrichment of the target bacteria using the MNPs. Compared with current biosensors for bacteria detection, this biosensor has shown obvious advantages, such as shorter time (because only one immunoreaction between the MNPs and the target bacteria was needed), easier extension (because only one antibody, not a pair of antibodies, was needed and the HRP could be changed by other enzymes or labels to develop new assays), and lower cost (because less expensive materials were used).

Besides, its specificity was evaluated using this biosensor to detect three other types of bacteria, such as *E. coli* O157:H7, *Listeria monocytogenes*, *Staphylococcus aureus*, and four other serotypes of *Salmonella*, such as *Salmonella* derby, *Salmonella* enteritidis, *Salmonella* mbandaka, and *Salmonella* meleagridis. Three parallel tests on these seven non-target bacteria at the concentration of 10^4^ CFU/mL were conducted. As shown in Figure 5c, the target bacteria has obviously higher separation efficiency (96.7%) and absorbance (0.34) than these three other types of bacteria (1.3%–1.6% for separation efficiency and 0.12–0.13 for absorbance) and these four other serotypes of *Salmonella* (4.1%–9.1% for separation efficiency and 0.13–0.17 for absorbance), indicating that this biosensor is specific for *Salmonella* typhimurium.

### 3.6. Detection of Salmonella Typhimurium in Spiked Apple Juice

To further evaluate the applicability of this biosensor for detection of *Salmonella* typhimurium in real samples, apple juice was purchased from local supermarket and first confirmed without target *Salmonella* using conventional culture plating. Then, different concentrations of *Salmonella* typhimurium were added into the juice to prepare the spiked sample with the bacterial concentrations from 1.4 × 10^2^ to 1.4 × 10^6^ CFU/mL. Three parallel tests on each concentration of the spiked juice were finally conducted using this biosensor. The recovery for each concentration of the target bacteria was calculated as the ratio of the detected concentration to the spiked one and shown in Table 1. The recovery ranged from 83% to 124% with the average recovery of 102%. This verified the applicability of this biosensor for detecting *Salmonella* typhimurium in real samples.

## 4. Conclusions

In this study, we successfully developed a microfluidic biosensor based on viscoelastic particle separation and enzyme catalytic colorimetry for rapid and sensitive detection of Salmonella typhimurium. The biosensor was able to detect Salmonella typhimurium as low as 30 CFU/mL within 1 h. This particle separation method based on viscoelastic inertial microfluidics was demonstrated to successfully separate the magnetic HRP-bacteria at the size of micrometer from the unbound MNPs at the size of nanometer. Compared with some reported biosensors for bacteria detection in Table S1, this biosensor has obvious merits of shorter time and/or higher sensitivity. However, it still needs further efforts to integrate magnetic separation of the target bacteria from sample, viscoelastic separation of the magnetic bacteria from the unbound MNPs and colorimetric detection of the catalysate onto a single chip to achieve fully automatic detection.

## Figures and Tables

**Table 1:** Detection of *Salmonella* typhimurium in apple juice using this biosensor (N = 3).

Spiked Conc. (CFU/mL)	Absorbance	Detected Conc. (CFU/mL)	Recovery	CV
0	0.128	ND ^a^	-	-
138	0.231	171	124%	1.5%
1380	0.306	1144	83%	1.0%
13,800	0.401	12,284	89%	0.5%
138,000	0.497	135,543	98%	0.7%
1,380,000	0.595	1,588,333	115%	1.8%

^a^ ND: Not Detectable.

## Supplementary Materials

The following are available online at https://www.mdpi.com/1424-8220/20/9/2738/s1, Figure S1: Photo of the microfluidic channel; Sketch of the microchannel, Table S1: Compared with some reported biosensors for bacteria detection.
