# Supplementary material for: A Rapid and Sensitive Salmonella Biosensor Based on Viscoelastic Inertial Microfluidics
Source: Sensors (Basel). 2020 May 11;20(9):2738. doi: 10.3390/s20092738 (PMC7248794; doi:10.3390/s20092738)
Supplement: Supplementary file 1 [file sensors-20-02738-s001.pdf]

Article

A Rapid and Sensitive *Salmonella* Biosensor Based on Viscoelastic Inertial Microfluidics

Lan Yao, Lingyan Zheng, Gaozhe Cai, Siyuan Wang, Lei Wang and Jianhan Lin*

Key Laboratory of Agricultural Information Acquisition Technology, Ministry of Agriculture and Rural Affairs, China Agricultural University, Beijing 100083, China; ylan2014@cau.edu.cn (L.Y.); Lingyanzheng@cau.edu.cn (L.Z.); gaozhe@cau.edu.cn (G.C.); wangsiyuan@cau.edu.cn (S.W.); wanglei123@cau.edu.cn (L.W.)

* Correspondence: jianhan@cau.edu.cn

Received: 9 April 2020; Accepted: 9 May 2020; Published: 11 May 2020

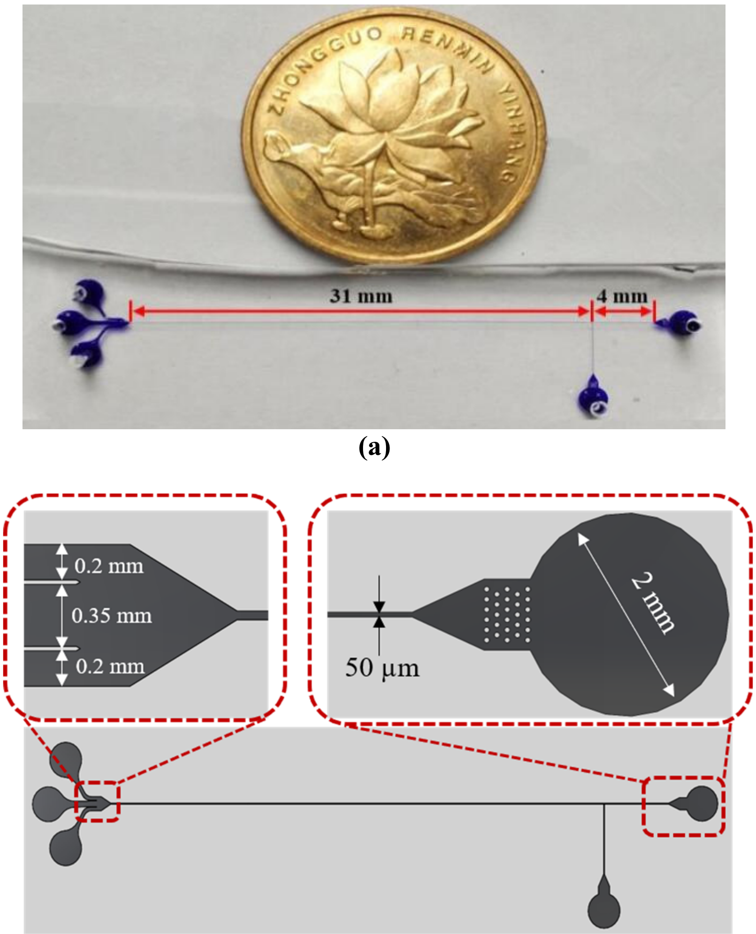

(a)

(b)

Figure S1. (a) Photo of the microfluidic channel; **(b)** Sketch of the microchannel.

Table S1. Compared with some reported biosensors for bacteria detection.

Method	Detection time	Used Antibodies*	Linear range (CFU/mL)	LOD (CFU/mL)	Ref.
Photothermal effect	1.5 h	Ab_1	300– 1000	300	[1]
Fluorescence	1 h	Ab_1, Ab_2	10^0–10^7	10^3	[2]
Electrochemical impedance	1 h	Ab_1	10^2–10^7	10^2	[3]
Colorimetry	50 min	Ab_1, Ab_2	10^3–10^6	10^3	[4]
ELISA	4 h	Ab_1, Ab_2	10^2–10^4	1	[5]
Colorimetry	15 min	Ab_1	10^3–10^5	10^4	[6]
Magnetophoretic chromatography	1 h	Ab_1	10^1–10^5	10^2	[7]
Colorimetry	1 h	Ab_1	10^2–10^6	30	This work

* Ab_1: one kind of antibody; Ab_2: the other kind of paring antibody.

References

1. Zhang, Z.; Wang, Q.; Han, L.; Du, S.; Yu, H.; Zhang, H. Rapid and sensitive detection of Salmonella typhimurium based on the photothermal effect of magnetic nanomaterials. *Sens. Actuators B Chem.* **2018**, *268*, 188–194, doi:10.1016/j.snb.2018.04.043.
2. Xu, L.; Lu, Z.; Cao, L.; Pang, H.; Zhang, Q.; Fu, Y.; Xiong, Y.; Li, Y.; Wang, X.; Wang, J.; et al. In-field detection of multiple pathogenic bacteria in food products using a portable fluorescent biosensing system. *Food Control* **2017**, *75*, 21–28, doi:10.1016/j.foodcont.2016.12.018.
3. Li, Z.; Fu, Y.; Fang, W.; Li, Y. Electrochemical Impedance Immunosensor Based on Self-Assembled Monolayers for Rapid Detection of Escherichia coli O157: H7 with Signal Amplification Using Lectin. *Sensors* **2015**, *15*, 19212–19224, doi:10.3390/s150819212.
4. Farka, Z.; Cunderlova, V.; Horackova, V.; Pastucha, M.; Mikusova, Z.; Hlavacek, A.; Skladal, P. Prussian Blue Nanoparticles as a Catalytic Label in a Sandwich Nanozyme-Linked Immunosorbent Assay. *Anal. Chem.* **2018**, *90*, 2348–2354, doi:10.1021/acs.analchem.7b04883.
5. Zeinhom, M.M.A.; Wang, Y.; Sheng, L.; Du, D.; Li, L.; Zhu, M.-J.; Lin, Y. Smart phone based immunosensor coupled with nanoflower signal amplification for rapid detection of Salmonella Enteritidis in milk, cheese and water. *Sens. Actuators B Chem.* **2018**, *261*, 75–82, doi:10.1016/j.snb.2017.11.093.
6. Chen, Y.; Xianyu, Y.; Sun, J.; Niu, Y.; Wang, Y.; Jiang, X. One-step detection of pathogens and cancer biomarkers by the naked eye based on aggregation of immunomagnetic beads. *Nanoscale* **2016**, *8*, 1100–1107, doi:10.1039/c5nr07044a.
7. Kwon, D.; Joo, J.; Lee, J.; Park, K.H.; Jeon, S. Magnetophoretic chromatography for the detection of pathogenic bacteria with the naked eye. *Anal. Chem.* **2013**, *85*, 7594–7598, doi:10.1021/ac401717f.
